# High Social Anxiety and Poor Quality of Life in Patients With Pulmonary Tuberculosis

**DOI:** 10.1097/MD.0000000000000413

**Published:** 2015-01-26

**Authors:** Erkan Kibrisli, Yasin Bez, Ahmet Yilmaz, Hamza Aslanhan, Mahsuk Taylan, Halide Kaya, Abdullah Cetin Tanrikulu, Ozlem Abakay

**Affiliations:** From the Department of Family Medicine (EK, AY, HA), School of Medicine, Dicle University, Diyarbakir; Department of Psychiatry (YB), School of Medicine, Marmara University, Istanbul; and Department of Chest Diseases (MT, HK, ACT, OA), School of Medicine, Dicle University, Diyarbakir, Turkey.

## Abstract

Pulmonary tuberculosis (PT) has been previously related with various psychosocial adverse consequences including stigmatization and social isolation.

Social anxiety is a psychiatric condition that may be associated with social isolation and fear of social exclusion.

To date no study has investigated social anxiety and its impact on quality of life (QoL) among patients with PT. Therefore, we aimed to determine the severity of social anxiety in a group of patients with PT.

Among patients who were recently discharged from hospital with the diagnosis of PT 94 patients and 99 healthy control subjects who had similar demographical features have been included in the study. A psychiatrist interviewed all participants and a semistructured interview form, which was prepared by the authors, Liebowitz Social Anxiety Scale (LSAS), and Short Form-36 were administered to them.

Patients with PT showed higher levels of performance avoidance and social avoidance than healthy control subjects. They reported lower QoL scores across all dimensions. Among patients women showed higher levels of LSAS subscale scores and total score. Fear of social exclusion was predicted by perceived illness severity and emotional role difficulty. On the other hand, perceived illness severity was predicted by fear of exclusion and sedimentation level.

PT patients seem to experience higher levels of social anxiety and associated fear of social exclusion that add to their worse QoL during the earlier months of their disease. Among them fear of social exclusion is related with perceived illness severity.

## INTRODUCTION

Pulmonary tuberculosis (PT) is a contagious and debilitating disease with many adverse consequences. Various psychosocial conditions including depression, anxiety, feelings of loneliness, feeling stigmatized, social isolation, and poor quality of life (QoL) have been previously reported among these patients.^1,2^ Moreover, in a recent study PT patients were reported to have lower QoL levels despite a proper antituberculosis treatment.^3^

Social anxiety is a condition in which an individual feels ashamed or humiliated at various social settings. Patients with social anxiety usually avoid these situations due to their excessive concerns.^4^ Also, they commonly isolate themselves from social interactions, which provoke their anxiety and concerns.

Social anxiety associated with various disfiguring or disabling medical conditions has been studied in recent years.^5–9^ These studies have concluded that the conditions were associated with a significant amount of distress in the effected patients. Physical and emotional confidences, in addition to social acceptance, are important aspects of the normal socialization process. PT is a disabling medical condition that may interfere with the sense of confidence both physically and emotionally in social settings. On the other hand, because it is historically known to be contagious and life-threatening, social acceptance of patients with tuberculosis may be compromised widely in society. As a consequence, they may feel isolated from social interactions due to the overt or covert behaviors or ideas of both themselves and the remaining society.^10^ Thus, one may expect that patients with tuberculosis may experience symptoms of social anxiety and general avoidance of activities to some extent.

Although various aspects of psychosocial status of patients with PT have been previously studied, to the best of our knowledge the severity of social anxiety that they experience has not been thoroughly studied. Therefore, we aimed to determine the severity of social anxiety in a group of patients with pulmonary parenchymal tuberculosis. Additionally, we aimed to compare the disease-related QoL of these patients with that of healthy controls.

## MATERIALS AND METHODS

### Study Population

A total of 110 patients who were hospitalized in Dicle University hospital, a pulmonary medicine inpatient clinic, which is at the Diyarbakir province of Turkey, between January 2013 and December 2013 who were recently diagnosed with pulmonary parenchymal tuberculosis were screened for the study 1 month after their discharge, and their willingness to participate was determined. Among them 94 patients (45 females, 49 males) with a mean age of 31.7 ± 13.8 years (range 15–64 years) met inclusion criteria, agreed to participate, and provided written informed consent. Each patient had a complete physical examination and was tested for acidoresistant bacilli positivity in their sputum. The inclusion criteria for patients were being over 18 years of age, at least a primary school graduate, and willing to participate in the study. Patients were excluded if they had a current or past history of any chronic medical or psychiatric condition, were taking any medication for any purposes other than tuberculosis, and had any other disfiguring or disabling condition that might cause psychological distress.

The control group in this study was composed of 99 healthy subjects (51 females, 48 males) who were similar to the patient group in terms of demographic variables including age, gender, and education with a mean age of 30.4 ± 11.3 years (range 18–64 years). Presence of any signs or evidence of tuberculosis in the control subjects led to their exclusion from the study. Subjects with known current or past disfiguring or disabling condition that may cause psychological distress were also excluded from the study. The institutional local ethics committee approved the study protocol. All control subjects gave informed consent to participate in the study. We have the local ethics committee's approval document.

### Data Collection and Measurements

#### Interview Form

This was prepared by the authors to collect demographic variables including age, gender, educational level, and marital status of the participants and to determine patients’ attitudes toward their diagnosis. Additionally, 2 visual analogue scales designed to measure perceptions of the patients regarding their illness severity and how anxious they feel given the possibility to be excluded from society (fear of social exclusion) were included in this form.

#### Assessment of Perceived Disease Severity and Fear of Social Exclusion

Authors of the present study used a visual analogue scale to assess the severity of disease among PT patients and fear of social exclusion. They drew a 10 cm long horizontal line with one end representing “no disease burden” and the other end “most severe disease that can ever happen” for assessing perceived disease severity. A similar 10 cm horizontal line was used to assess their fear of social exclusion where end points are labeled “no fear of social exclusion” and the “most extreme fear of social exclusion that can ever happen.” Patients were asked to locate their disease severity and fear of social exclusion on these lines separately. Patients crossed the lines only at one point where they considered their representative disease severity or fear of social exclusion. The extremes were assigned numeric values 100 and 0, respectively. Higher scores reflect worse disease severity and increased levels of social exclusion fear, respectively. Such questionnaires have already been used in previous studies to assess subjective sensations or perceptions of patients.^11–13^

#### Liebowitz Social Anxiety Scale (LSAS)

The LSAS is a questionnaire first developed by Michael R. Liebowitz for assessing the severity of fear and avoidance in social interactions and performance situations.^14^ Its reliability, validity, and treatment sensitivity were shown previously in social phobia patients.^15^ It helps to quantify the severity of social phobia symptoms; however, it cannot be used as a diagnostic tool. The questionnaire includes 24 items, 11 assessing social situations and 13 assessing performance situations. Administered by a clinician, the scale provides scores on 6 subscales, measuring the severity of fear in social situations, the severity of performance fear, the severity of social avoidance, the severity of performance avoidance, the severity of total fear, and the severity of total avoidance. Validity and reliability of the Turkish version of LSAS has been previously demonstrated.^14^

#### Short Form-36 (SF-36)

SF-36 is a widely used self-report scale to measure health-related QoL. It successfully measures patients with medical or psychological disorders and healthy subjects as well. Positive and negative aspects of health can be assessed by it and it is accepted to be sensitive to small changes in disability status. Ware and Sherbourne first developed it in 1992.^16^ Higher scores reflect a better QoL. The validity and reliability of the Turkish version of SF-36 was demonstrated by Kocyigit et al in 1999.^17^

### Procedure

The interview form prepared by the authors and the patient charts were used to gather the data about the demographic and clinical features of the patients and the control group. An experienced psychiatrist (Y.B.) administered the LSAS to assess the severity of social anxiety and avoidance in patient and control groups. The patients and the control group completed a generic measurement to evaluate health-related QoL (SF-36).

### Statistical Analysis

The statistical analysis of the data was done by the Statistical Package for Social Sciences 11.5 software. Normality of data has been tested with Kolmogorov–Smirnov test. The comparison of the continuous variables was accomplished by Student *t* test, and for the comparison of the categorical variables the χ^2^ test was used. The Pearson correlation analysis was used in the evaluation of the correlation between scores of the different scales and other relevant variables. Multiple linear regression analyses have been run to test the significant predictors of fear of social exclusion and perceived illness severity. The continuous variables in the entire article have been presented in the form of mean ± standard deviation (SD). *P* values <0.05 are accepted as statistically significant.

## RESULTS

Demographic variables of both patient and control groups such as age, gender distribution, and education level are shown in Table 1. Both groups were statistically similar in terms of demographic variables. The patient group was composed of relatively young adults given their mean age (31.7 ± 13.8 years). Most of the patients reported loss of appetite (93.6%, n = 88), weight loss (91.5%, n = 86), and night sweats (83%, n = 78) as their symptoms. All the patients were new cases of PT who were initially hospitalized and discharged after planning of their management.

**Table 1:**
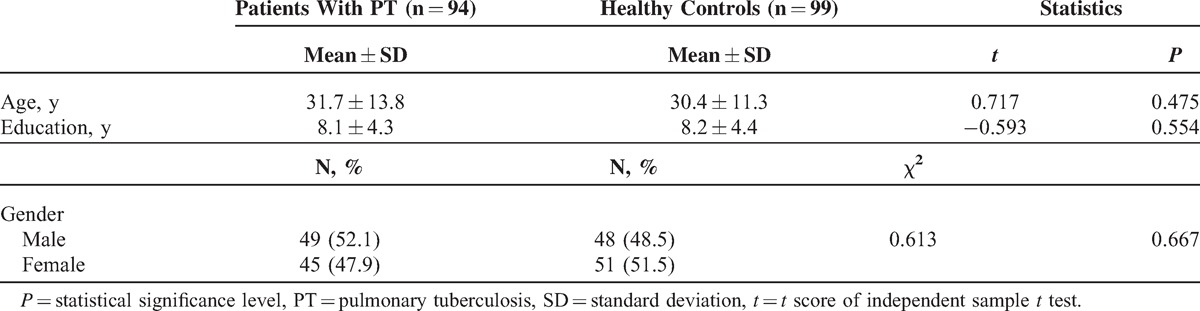
Comparison of Demographic Variables of Patients and Control Subjects

When patients and control subjects were compared in terms of levels of their social anxiety symptoms and QoL, there were some statistically significant differences. Patients with tuberculosis showed significantly higher levels of performance avoidance, social avoidance, total avoidance, and LSAS total score than control subjects. Patients have reported significantly lower scores in all subscales of SF-36 that suggests a poorer QoL in PT patients in all dimensions measured by the scale than that in control subjects (Table 2).

**Table 2:**
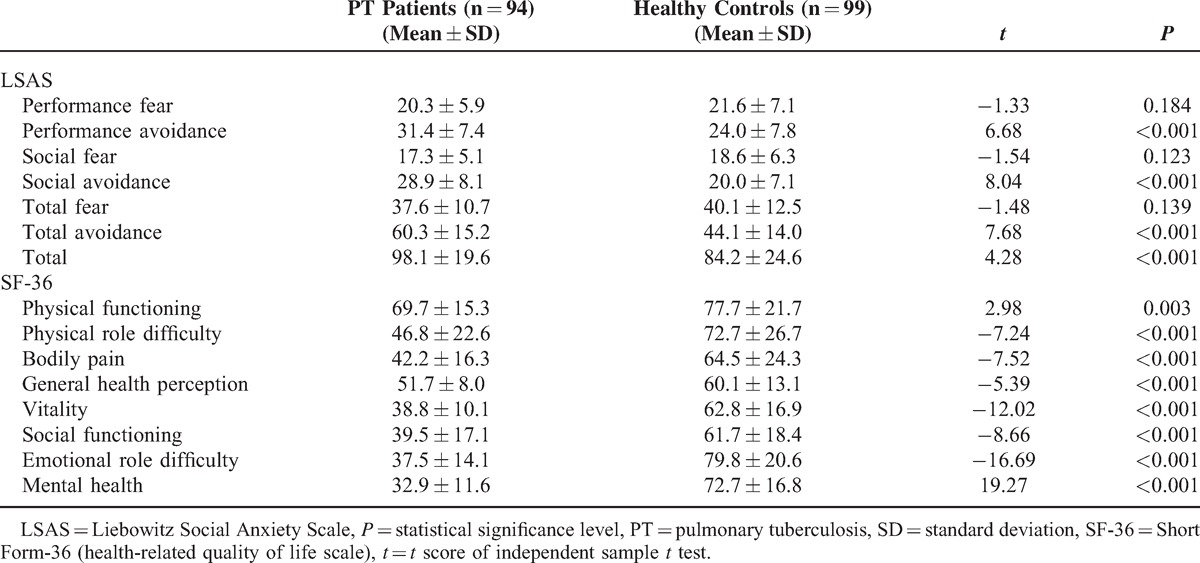
Mean LSAS and SF-36 Scores of PT Patients and Healthy Control Subjects

In the patient group, genders were compared in terms of study variables including subjective levels of perceived illness severity and fear of social exclusion, severity of social anxiety symptoms, and QoL scale. Among them only LSAS subscale scores and total score were significantly higher in women when compared with those in men (Table 3).

**Table 3:**
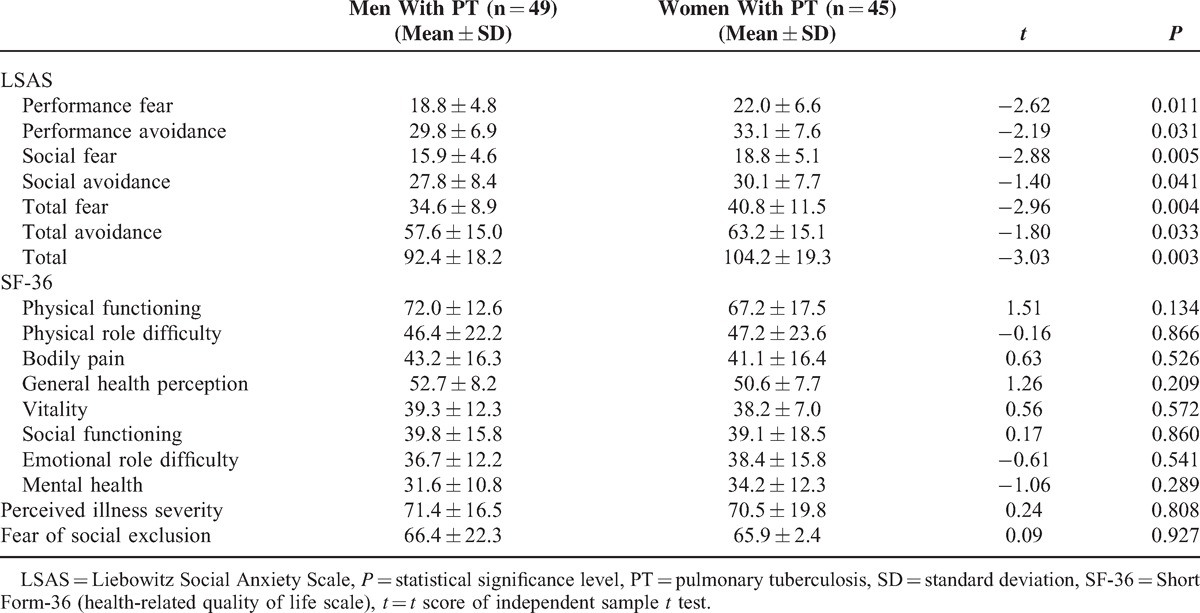
Mean LSAS and SF-36 Scores, Perceived Illness Severity, and Fear of Social Exclusion Levels of Both Genders and Their Comparisons

The fear of social exclusion (mean ± SD: 66.2 ± 23.7, minimum–maximum: 2–96) in the patients was positively correlated with perceived illness severity, performance avoidance, social avoidance, total avoidance scores, and total score of LSAS. It was negatively correlated with albumin level, social functioning, emotional role difficulty, and mental health. Among many linear regression models tested, the best-fit model used fear of social exclusion as a dependent variable and the above-mentioned variables as the independent variables (F = 10.26, df = 11, *R*^2^ = 0.57, adjusted *R*^2^ = 0.52, *P* < 0.001). Perceived illness severity and emotional role difficulty significantly predicted the fear of social exclusion (B = 0.375, *P* < 0.001; B = −0.362, *P* < 0.001, respectively).

Mean perceived illness severity was 70.9 ± 18.1 (minimum–maximum: 2–99). It was positively correlated with fear of social exclusion, performance and total avoidance scores of LSAS, and sedimentation level, whereas social functioning and mental health were correlated negatively with this parameter. Many linear regression analyses using perceived illness severity as a dependent variable have been conducted. The best-fit model included fear of social exclusion, performance and total avoidance scores of LSAS, sedimentation, social functioning, and mental health as independent variables (F = 7.16, df = 6, *R*^2^ = 0.33, adjusted *R*^2^ = 0.28, *P* < 0.001). Fear of exclusion and sedimentation levels were found to be significant predictors of perceived illness severity (B = 0.536, *P* < 0.001; B = 0.204, *P* = 0.024, respectively).

Hemoptysis may be considered a significant symptom of Tuberculosis (TB) that is often assumed to be an emergent signal by the patients who are affected. It was present in 37.2% (n = 35) of the patients with TB. Therefore, we compared the psychological status and QoL of patients with and without hemoptysis. This comparison revealed no difference in terms of study variables.

## DISCUSSION

To our knowledge this is the first study that investigated social anxiety, fear of exclusion, and perceived illness severity among patients with pulmonary parenchymal TB. In this study, social anxiety severity and QoL of the patients were compared with those of healthy control subjects. In addition, fear of exclusion and perceived illness severity and correlates of them were studied in these patients. Patients with TB showed increased levels of performance avoidance and social avoidance than healthy control subjects. Also, they reported worse QoL in all dimensions as determined by SF-36, which is a generic, widely known, and reliable health-related QoL measurement. Both groups were similar in terms of age, gender, and level of education; therefore, at first glance it is hard to attribute the above-mentioned differences to these similarities.

In recent years, a topic of interest in TB patients has been self-discrimination, isolation, and stigmatization.^18,19^ Traditionally it is known that TB patients feel that they are excluded from the population due to concerns mainly related to disease dissemination.^20,21^ Many studies have demonstrated that stigma is not a subjective feeling of the affected patients per se but that the people around them really do behave in a way that isolates them.^21,22^ Stigma as a socially constructed phenomenon can shape the attitudes and behaviors of others toward those affected by the disease.^10^ In some previous studies, feelings of shame, embarrassment, or social isolation have been reported among TB patients.^23^ However, social anxiety, a psychiatric condition mainly characterized by these feelings, among them has not been thoroughly investigated, yet. In a previous review focusing on stigma of tuberculosis, authors concluded that stigma accompanying TB could have a negative impact on the individual and family, which may result in their withdrawal from society because of shame and fear.^2^ Consistent with these previous reports our study has shown that patients with PT experience higher levels of social anxiety in the form of basic avoidance. Thus, one may think that concerns about disease dissemination may be associated with their avoidance.

Since stigmatization in TB patients may limit their socialization, it is hard to differentiate the effects of stigmatization from the symptoms of social phobia. In both conditions, patients would make an effort to avoid conversations and feel distant from others. From the point of difficulty that the patients experience they are very similar and seem to be interwoven. Thus, clinicians should keep in mind that both may exacerbate social anxiety symptoms of the patients and in some occasions each may hinder appropriate management of the other.

Women in our study were more anxious socially than men. One may think that concerns about disease dissemination may be associated with their avoidance. The positive correlation observed between avoidance scores and their subjective fear of social exclusion provides some additional support to this suggestion. It may be worthwhile to mention that half of the variance in subjective fear of social exclusion in our patients was predicted by perceived disease severity and emotional role difficulty.

PT patients in our study showed worse QoL in all dimensions than healthy control subjects. Similarly, lower scores across all SF-36 subscales in active TB patients have been reported in the literature.^24^ Although improvement in objective laboratory findings is used as a major outcome measure in disease management, today, patient-reported outcomes such as health-related QoL have also become valuable in daily clinical practice. In our study, a generic instrument to determine QoL was used since a validated TB-specific measure has not been developed yet. In some studies that used SF-36 to assess the QoL in TB patients it is clearly shown that TB patients have poor QoL that persists even after treatment.^25^ A number of previous studies have demonstrated a gender effect on QoL among TB patients that favors men over women.^26,27^ Contrary to their findings, our results did not show any difference between genders in terms of QoL. However, different from their study populations, our patients were all hospitalized. A study similar to ours found that hospitalized TB patients reported no significant association between gender and QoL.^28^ Among common symptoms of TB, hemoptysis attracts special attention of both clinicians and patients. Although a poorer QoL has been suggested with the development of hemoptysis, our data did not prove this, at least for the hospitalized patients with PT.^24^

To the best of our knowledge, current literature is lacking a verified scoring system to determine the severity of TB. With the hope to overcome this issue we used a 10 cm horizontal line (a visual analogue scale) to determine the perceived severity of TB. The line represented “no burden of disease” at one end and “most severe disease that can ever happen” at the other end. Patients were asked to cross the 10 cm line only at one point where they consider to be showing the severity of their disease. The patients in our study can be considered to be moderately ill when subjectively evaluated with the visual analogue scale described above. Their perceived illness severity was positively correlated with the severity of fear of social exclusion, performance and total avoidance scores, and sedimentation level, whereas it was negatively correlated with social functioning and mental health. Among these correlating variables, sedimentation is the only objective factor that can easily be measured in a laboratory setting. Moreover, regression analysis in the present study showed that it can be considered a significant predictor of perceived illness severity in pulmonary parenchymal TB patients. In clinical practice, higher sedimentation levels are considered to point out a more severe disease in regards to TB. When taken together, sedimentation seems to reliably reflect perceived illness severity in PT patients or vice versa.

The present study has some limitations. First, effects of PT diagnosis on social anxiety level and QoL of patients would be better understood if we were able to determine their premorbid levels. Trying to determine these levels retrospectively would not be reliable since it may harbor recall bias. Therefore, to overcome this limitation we have used a control group similar to the patient group in terms of demographic variables. Second, results of this study may represent only the first month following a PT diagnosis; thus, they do not reflect the long-term status of the patients. Third, we were able to test the effects of only hemoptysis among some specific clinical symptoms because frequency of other symptoms including cough, weight loss, and night sweats did not allow us to conduct reliable statistical analyses.

In conclusion, PT patients experience higher levels of social anxiety and fear of social exclusion that accompany lower levels of QoL during the earlier months of their disease. Subjective fear of social exclusion seems to be related with perceived illness severity of these patients. Further studies demonstrating longer follow-up periods are needed.
